# Co-Administration of Vonoprazan, Not Tegoprazan, Affects the Pharmacokinetics of Atorvastatin in Healthy Male Subjects

**DOI:** 10.3389/fphar.2021.754849

**Published:** 2021-11-11

**Authors:** Sejung Hwang, Jae-Wook Ko, Heechan Lee, Seokuee Kim, Bongtae Kim, Geun Seog Song, Jungryul Kim

**Affiliations:** ^1^ Department of Clinical Pharmacology and Therapeutics, Seoul National University College of Medicine and Hospital, Seoul, South Korea; ^2^ Department of Clinical Pharmacology and Therapeutics, Samsung Medical Center, Seoul, South Korea; ^3^ Division of Clinical Development, HK Inno.N Corporation, Seoul, South Korea; ^4^ Department of Clinical Research Design and Evaluation, SAIHST, Sungkyunkwan University, Seoul, South Korea

**Keywords:** drug interactions, tegoprazan, vonoprazan, cytochrome P450 (CYP), pharmacokinetics, potassium-competitive acid blocker

## Abstract

Potassium-competitive acid blocker is a new class of drugs inhibiting gastric acid. It is controversial that vonoprazan showed the inhibitory activities of cytochrome P450 3A4. This study aimed to evaluate the pharmacokinetics (PK) of atorvastatin and safety when atorvastatin was administered alone and co-administered with vonoprazan or tegoprazan. An open-label, multiple-dose, 3-intervention, 4-sequence, 4-period, partial replicate crossover study was conducted, and three interventions were; one is orally administered atorvastatin 40 mg alone once daily for 7 days, another is atorvastatin co-administered with vonoprazan 20 mg, and the other is atorvastatin co-administered with tegoprazan 50 mg. PK blood samples were collected up to 24 h after the last dose, and PK parameters for atorvastatin, 2-hydroxyatorvastatin and atorvastatin lactone were estimated by a non-compartmental method. Safety was evaluated, including adverse events and clinical laboratory tests. A total of 28 subjects completed the study. When atorvastatin was co-administered with vonoprazan, the systemic exposures of atorvastatin and atorvastatin lactone significantly increased, and the metabolic ratio of 2-hydroxyatorvastatin significantly decreased. Hypergastrinemia only occurred when atorvastatin was co-administered with vonoprazan. However, the plasma concentration profiles of atorvastatin, 2-hydroxyatorvastatin and atorvastatin lactone were similar when atorvastatin was administered alone or co-administered with tegoprazan. In conclusion, after multiple doses of atorvastatin co-administered with vonoprazan in healthy subjects, the systemic exposure of atorvastatin and the incidence of hypergastrinemia increased. With tegoprazan, however, those interactions were not observed.

## Introduction

Proton pump inhibitors (PPIs) are commonly prescribed as the first-line drug for the treatment of acid-related diseases such as gastroesophageal reflux disease (GERD) by inhibiting H+/K + -ATPase (Katz et al., 2013; Otake et al., 2016). However, some limitations prevent PPIs from becoming the ideal antisecretory drugs. The major limitations are as follows: 1) PPIs are acid labile, 2) PPIs shows a slow onset of action, taking 3–5 days for the appropriate effect, 3) PPIs show interindividual variations in pharmacokinetics (PK) according to cytochrome P450 (CYP) 2C19, and 4) PPIs fail to suppress nocturnal acid secretion expected by the short half-life (Otake et al., 2016).

Potassium-competitive acid blocker (P-CAB) is a new class of drugs that inhibits gastric H+/K + -ATPase (Inatomi et al., 2016). While PPIs require chemical conversion to their active form and only inhibit an activated form of H+/K + -ATPase, P-CAB does not require acid activation and reversely inhibits both an activated and inactivated form of H+/K + -ATPase by competitively blocking the potassium-binding site (Mori and Suzuki, 2019). Therefore, P-CAB shows a rapid onset of acid suppression and continuous acid suppression until the night compared to PPIs (Okuyama et al., 2017; Mori and Suzuki, 2019; Cho et al., 2020).

Vonoprazan, a P-CAB developed by Takeda Pharmaceutical Company, received approvals for acid-related diseases in some countries, including Japan and Korea (Inatomi et al., 2016). Tegoprazan, a P-CAB developed by HK inno.N, was recently approved to treat acid-related diseases such as GERD (Lee et al., 2019). Vonoprazan and tegoprazan are mainly metabolized by CYP3A4 and partially by CYP2B6, 2C19, 2D6.

A recent study suggested the potential that vonoprazan acts as a moderate inhibitor of CYP2C19 and CYP3A4 (Otake et al., 2016; Kagami et al., 2018). The results of *in vitro* studies indicated that vonoprazan could inhibit the metabolism of midazolam, the substrate of CYP3A4, and venlafaxine, the substrate of CYP3A4 and CYP2D6. In addition, the *in vivo* studies supported the results of the *in vitro* studies, which implicated the drug-drug interactions between vonoprazan and the substrates of CYP3A4 (Chen et al., 2020; Wang et al., 2020). The half-maximal inhibitory concentration (IC50) value of CYP3A4 for vonoprazan was 29 μM, and this value is thought to be much higher than the plasma concentration of vonoprazan at clinical doses (Nishihara et al., 2019). However, the potential CYP3A4 inhibitory effect of vonoprazan on drugs having first-pass effect is unknown. Although tegoprazan showed various inhibitory activities to organic anion transporting polypeptides (OATP) 1B1 according to the *in vitro* study, the systemic exposure of the substrates would not significantly increase considering the maximum concentration of tegoprazan at clinical doses (HK inno.N Corp).

One of the substrates of CYP3A4 and OATP1B is 3‐hydroxy‐3‐methylglutaryl co‐enzyme A (HMG‐CoA) reductase inhibitors, including atorvastatin (US Food and Drug Administration, 2020a). The bioavailability of atorvastatin is approximately 14%, and this low bioavailability is attributed to first-pass metabolism in the intestinal mucosa and the liver, in which CYP3A4 is involved (Lennernäs, 2003). When absorbed through the intestine, 76% of orally administered atorvastatin is lost in the gut, and 42% of the remaining amount is lost in the liver (Lennernäs, 2003). Therefore, when atorvastatin was co-administered with a potent CYP3A4 inhibitor such as itraconazole, the systemic exposure of atorvastatin increased, and the systemic exposure of 2-hydroxyatorvastatin, a metabolite of atorvastatin, decreased (Teemu Kantola, 1998; Jacobson, 2004).

The number of PPI users increased 10.6 times over 12 years, and the prescription rate of atorvastatin generally increased over 13 years (Kim et al., 2018; Son and Bae, 2019). PPIs and HMG-CoA reductase inhibitors are frequently co-administered with clopidogrel in Korea (Kim et al., 2019). The frequency of co-administering PPIs and atorvastatin would increase over time, and thus it is necessary to evaluate the drug interactions between P-CABs and atorvastatin. This study aimed to evaluate the PK of atorvastatin and safety profile when co-administered with P-CABs, vonoprazan or tegoprazan.

## Materials and Methods

### Study Design and Interventions

This was an open-label, multiple-dose, 3-intervention, 4-sequence, 4-period, partial replicate crossover study (ClinicalTrials.gov Identifier: NCT04221321). The study protocol was approved by the institutional review board at Samsung Medical Center (Seoul, Republic of Korea) and the Ministry of Food and Drug Safety. All subjects gave written informed consent.

**FIGURE 1:**
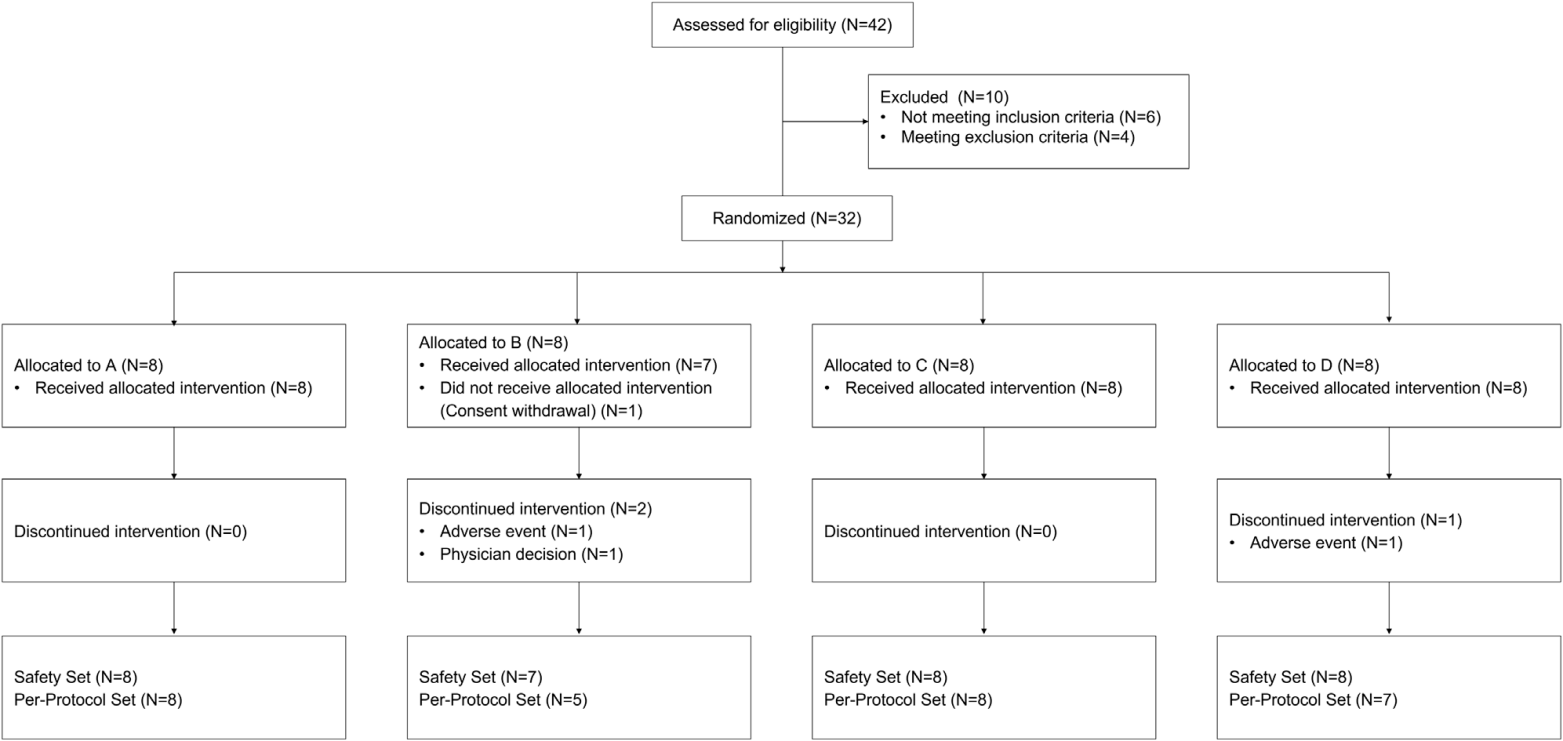
Subject disposition. A: sequence with intervention T-R1-V-R2, B: sequence with intervention R1-T-R2-V, C: sequence with intervention R1-V-R2-T, D: sequence with intervention V-R1-T-R2 (R1: the first intervention with the administration of atorvastatin alone once a day for 7 days, R2: the second intervention with the administration of atorvastatin alone once a day for 7 days, T: co-administration of atorvastatin and tegoprazan once a day for 7 days, V: co-administration of atorvastatin and vonoprazan once a day for 7 days).

There were three interventions in this study, and daily administration for 7 days of atorvastatin 40 mg (LIPITOR^®^, Pfizer Inc., NY, United States) alone was defined as intervention R. Moreover, atorvastatin 40 mg co-administered with tegoprazan 50 mg (K-CAB^®^, HK inno.N Corporation, Republic of Korea) and atorvastatin 40 mg co-administered with vonoprazan 20 mg (TAKECAB^®^, Takeda Pharmaceutical Company Limited, Japan) was named as intervention T and intervention V, respectively. Among three interventions, subjects received intervention R twice and received intervention T and V once each. Subjects were allocated to one of the four sequences: T-R-V-R, R-T-R-V, R-V-R-T, and V-R-T-R (Figure 1). There were washout periods of 7–9 days between each intervention. Furthermore, this study was carried out in three groups since entire subjects could not be studied simultaneously. Assuming the within-subject coefficient of variation (CV) of atorvastatin as 40 and 15% as dropout rate, the planned sample size was 36 subjects providing more than 80% statistical power with a significance level of 0.05 to demonstrate 33% difference of PK parameter by drug interactions (Tothfalusi and Endrenyi, 2012).

### Subjects

This study enrolled healthy male subjects aged 19–55 years with a body mass index of 19–27 kg/m^2^. Major exclusion criteria were: a history of genetic myopathy or family history; a history of taking any medications or foods that could significantly affect the PK of atorvastatin within 30 days before the randomization; blood aspartate aminotransferase, alanine aminotransferase or gamma-glutamyltransferase levels exceeding 1.5 times the upper limit of the reference range; an estimated glomerular filtration rate less than 60 ml/min/1.73 m^2^.

### Procedure and Assessments

Subjects had scheduled visits on the morning of Days 1–4 for dosing and then were admitted to the Clinical Trial Center of Samsung Medical Center on the evening of Day 4. Subjects received the study drug orally with water of 150 ml after an overnight fast of at least 10 h on Days 5–7 and were required to fast for an additional 4 h postdose on Day 7.

Serial blood samples were collected for PK assessment before dosing and at 0.25, 0.5, 0.75, 1, 1.25, 1.5, 2, 3, 4, 6, 9, 12, 15, and 24 h after the last repeated dose (Day 7). Blood samples were centrifuged at approximately 1,800 g for 10 min at 4°C, and then the plasma samples were stored at less than -70°C until the assays.

Safety was assessed based on symptom reports, physical examinations, vital signs, 12-lead ECGs, and clinical laboratory tests (hematology, clinical chemistry, and urinalysis).

### Assay of Atorvastatin and Its Metabolites

Plasma samples for atorvastatin, 2-hydroxyatorvastatin and atorvastatin lactone were analyzed with ultra‐performance liquid chromatography (Waters ACQUITY UPLCTM System; Waters, Milford, MA) - tandem quadruple spectrometry (Waters XevoTM TQ-XS MS; Waters, Milford, MA) method. Atorvastatin-d5, 2-hydroxyatorvastatin-d5 and atorvastatin lactone-d5 were used as the internal standard. The range for quantification was 0.100–200 ng/ml for atorvastatin, 0.0500–100 ng/ml for 2-hydroxyatorvastatin and atorvastatin lactone. The correction coefficients (r2) were greater than 0.99 over these concentration ranges. The precision and accuracy for mean within-run data and mean between-run data of all analytes were determined using the percentage of relative SD (%RSD) and the percentage of deviation of the mean from theoretical (%DMT).

The precision and accuracy for mean within-run data on atorvastatin in quality control (QC) samples (concentration range, 0.100–150 ng/ml) ranged from 0.6 to 2.7 and -1.6 to 1.5, respectively. The precision and accuracy for mean between-run data on atorvastatin in QC samples were determined from 1.1 to 2.5 and from −2.9 to −0.3, respectively. Based on back-calculated concentration, the between-run precision and accuracy ranged from 0.3 to 1.6 and from −1.8 to 2.1, respectively.

The within-run precision and accuracy for two metabolites in QC samples (concentration range, 0.0500–75.0 ng/ml) ranged from 0.4 to 4.6 and −3.0 to 0.7 on 2-hydroxyatorvastatin, respectively, and from 1.5 to 2.3 and 1.4 to 7.3 on atorvastatin lactone, respectively. The between-run precision and accuracy in QC samples were determined from 0.9 to 4.9 and −2.0 to -0.5 on 2-hydroxyatorvastatin, respectively, and from 2.1 to 2.8 and −1.9 to 5.0 on atorvastatin lactone, respectively. Based on back-calculated concentration, the between-run precision and accuracy were in a range of 0.5–2.4 and −0.8–0.8 on 2-hydroxyatorvastatin and 1.2–3.7 and -3.8–4.5 on atorvastatin lactone, respectively.

### Statistical Analysis

The PK profiles of atorvastatin and its metabolites were evaluated based on the following parameters: the maximum plasma concentration (C_ss,max_) and area under the concentration-time curve (AUC_τ_) at steady state for each analyte, time to C_ss_,_max_ (T_ss,max_) for atorvastatin, and metabolic ratio for atorvastatin metabolites. The PK parameters were obtained directly from the data or estimated by a non-compartmental method using MATLAB SimBiology (The MathWorks, Inc, 2020). The descriptive statistics for the PK parameters were summarized by the intervention, and the comparison of PK parameters was performed on C_ss,max_ and AUC_τ_. Log-transformed C_ss,max_ and AUC_τ_ values were assessed by a mixed-effects model. The model defined sequence, group, period nested in group and intervention as fixed effects and subject as a random effect, where the cluster of subjects studied at one time was reflected in the group effect. Intervention R was considered a reference in this analysis, and no adjustment was made for multiple comparisons unless otherwise specified. Subject characteristics and adverse events (AEs) were also summarized. The changes in serum gastrin, pepsinogen I and II levels were compared by mixed-effects model with Tukey-Kramer method. To explore the effect of co-administered with tegoprazan or vonoprazan on the low-density lipoprotein cholesterol (LDL-C) lowering effect of atorvastatin (Kenward et al., 2010), the changes of LDL-C were compared between the interventions by mixed-effects model and the post-hoc analysis was performed using Tukey-Kramer method. Statistical analysis was carried out using SAS Enterprise Guide (Version 7.1; SAS Institute Inc., NC, United States) with a significance level of 0.05.

## Results

### Study Population

A total of 32 subjects were enrolled, and 28 subjects completed the study (Figure 1). One subject withdrew the consent before the investigational product administration, and 3 subjects discontinued the study due to AEs (2 subjects) and the physician decision (1 subject). Twenty-eight subjects, who had complete PK profiles, were included for PK analysis and safety was analyzed in 31 subjects (31 subjects in intervention R1, 28 subjects in intervention R2, 30 subjects in intervention T, and 29 subjects in intervention V) who had received any intervention at least once. The mean (±standard deviation) values of age, weight, height, and body mass index (BMI) of the 32 enrolled subjects were 33.9 (±9.6) years, 71.1 (±8.1) cm, 174.6 (±6.0) kg, and 23.3 (±2.6) kg/m^2^. The demographic characteristics were similar among the allocation groups.

### Pharmacokinetics

**FIGURE 2:**
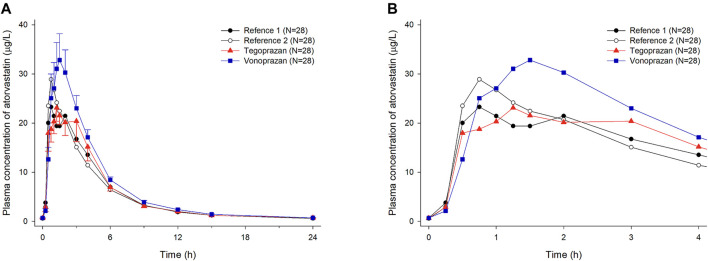
Mean plasma concentration-time profiles of atorvastatin after oral administration of atorvastatin alone for 7 days (Reference 1, 2) and co-administered with tegoprazan (Tegoprazan) or vonoprazan (Vonoprazan) for 7 days. Bars represent standard errors. [**(A)** linear scale and **(B)** up to 4 h after 7 days of oral administration].

Mean plasma concentration profiles of atorvastatin when atorvastatin was administered alone and co-administered with vonoprazan or tegoprazan are shown in Figure 2A. For better visual inspection of the absorption phase of atorvastatin, the mean plasma concentration profiles of atorvastatin up to 4 h are represented in Figure 2B.

**FIGURE 3:**
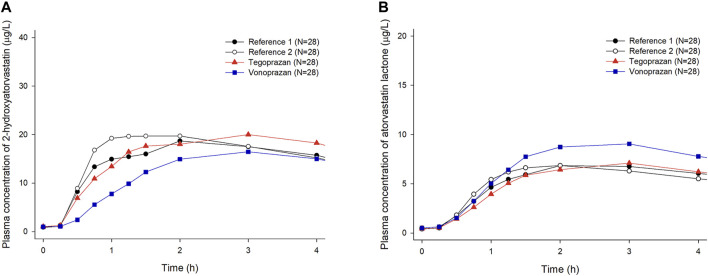
Mean plasma concentration-time profiles of 2-hydroxyatorvastatin and atorvastatin lactone up to 4 h after oral administration of atorvastatin alone once a day for 7 days (Reference 1, 2) and co-administered with tegoprazan (Tegoprazan) or vonoprazan (Vonoprazan) for 7 days. Bars represent standard deviations. [**(A)** 2-hydroxyatorvastatin, **(B)** atorvastatin lactone].

When atorvastatin was co-administered with vonoprazan, the C_ss_,_max_ of atorvastatin increased by 17% and AUC_τ_ of atorvastatin increased by 28% compared to those when atorvastatin was administered alone (Table 2). Although the systemic exposure of atorvastatin increased, the C_ss,max_ and AUC_τ_ of 2-hydroxyatorvastatin decreased by 30 and 9%, respectively, when atorvastatin was co-administered with vonoprazan compared to those when administered alone (Supplementary Table S1). The mean plasma concentration profiles up to 4 h showed that 2-hydroxyatorvastatin was formed more slowly than when atorvastatin was administered alone (Figure 3A). The C_ss,max_ and AUC_τ_ of atorvastatin lactone increased by 32 and 29% respectively when atorvastatin was co-administered with vonoprazan compared to those when atorvastatin was administered alone (Supplementary Table S1). The noticeable increase of C_ss,max_ of atorvastatin lactone was also observed in the mean plasma concentration profiles up to 4 h (Figure 3B). However, the metabolic ratio of atorvastatin lactone was similar between the intervention groups since the systemic exposure of atorvastatin lactone increased to a similar extent to that of atorvastatin (Table 1).

**TABLE 1:** Pharmacokinetic parameters of atorvastatin and metabolites (2-hydroxy atorvastatin, atorvastatin lactone) after oral administration of atorvastatin for 7 days administered alone and co-administered with tegoprazan or vonoprazan.

		Reference 1 (N = 28)	Reference 2 (N = 28)	Tegoprazan (N = 28)	Vonoprazan (N = 28)
Atorvastatin	T_ss,max_ (h)*	0.75 (0.50–6.00)	0.75 (0.50–3.00)	1.25 (0.50–3.00)	1.50 (0.50–4.00)
	C_ss,max_ (μg/L)	37.21 ± 19.65	37.37 ± 22.81	40.75 ± 21.52	42.71 ± 25.76
	AUC_τ_ (h·μg/L)	125.61 ± 67.08	124.24 ± 61.34	130.26 ± 71.17	159.78 ± 82.11
2-hydroxyatorvastatin	C_ss,max_ (μg/L)	24.95 ± 11.77	25.72 ± 17.15	26.34 ± 12.64	18.19 ± 12.16
	AUC_τ_ (h·μg/L)	152.04 ± 70.25	144.35 ± 58.71	156.46 ± 67.69	135.57 ± 64.05
	Metabolic ratio	1.27 ± 0.52	1.32 ± 0.55	1.31 ± 0.47	0.90 ± 0.32
Atorvastatin lactone	C_ss,max_ (μg/L)	8.12 ± 4.78	7.91 ± 4.9	8.34 ± 5.04	10.35 ± 5.46
	AUC_τ_ (h·μg/L)	61.07 ± 30.20	57.36 ± 28.51	59.62 ± 30.99	74.97 ± 32.03
	Metabolic ratio	0.52 ± 0.21	0.49 ± 0.20	0.51 ± 0.23	0.51 ± 0.20

Notes: Reference 1, the first intervention of atorvastatin administered alone; Reference 2, the second intervention of atorvastatin administered alone; Tegoprazan, co-administration of atorvastatin and tegoprazan once a day for 7 days; Vonoprazan, co-administration of atorvastatin and tegoprazan once a day for 7 days.

Values are presented as mean ± standard deviation. The metabolic ratio was calculated by AUC_metabolite_/AUC_atorvastatin._

*Values are presented as median (minimum-maximum).

Abbreviations; AUC_τ_, area under the curve over a dosing interval at steady state; C_ss,max_, maximum concentration at steady state; T_ss,max_, time of Cmax at steady state.

When atorvastatin was co-administered with tegoprazan, the C_ss,max_ and AUC_τ_ of atorvastatin were similar to those when atorvastatin was administered alone (Table 1, Table 2). Moreover, there was no difference in the mean plasma concentration profiles of atorvastatin up to 4 h (Figure 2B). The C_ss,max_ and AUC_τ_ of 2-hydroxyatorvastatin and atorvastatin lactone were similar regardless of the tegoprazan co-administration (Supplementary Table S1).

**TABLE 2:** Comparison of pharmacokinetic parameters of atorvastatin after oral doses of atorvastatin for 7 days administered alone and co-administered with tegoprazan or vonoprazan.

Parameters	Geometric least squares mean		GMR (90% CI)	Geometric least squares mean		GMR (90% CI)
	With Tegoprazan (N = 28)	Atorvastatin alone (N = 28 for each intervention)		With Vonoprazan (N = 28)	Atorvastatin alone (N = 28 for each intervention)	
C_ss,max_ (μg/L)	35.4	32.4	1.09 (0.97–1.23)	37.9	32.4	1.17 (1.04–1.32)^*^
AUC_τ_ (h·μg/L)	114.9	112.0	1.03 (0.98–1.08)	143.4	112.0	1.28 (1.22–1.34)^*^

Tegoprazan, co-administration of atorvastatin and tegoprazan once a day for 7 days; Vonoprazan, co-administration of atorvastatin and tegoprazan once a day for 7 days.

GMR is calculated as a ratio of atorvastatin co-administered with tegoprazan or vonoprazan to administered alone.

*According to the equivalence test, if the 90% confidence interval is not included in the predefined equivalence range (0.8–1.25), it is not considered equivalent.

Abbreviations: AUC, area under the curve over a dosing interval at steady state; C_ss,max_, maximum concentration at steady state; CI, confidence interval; GMR, the geometric mean ratio.

### Safety and Tolerability

A total of 42 treatment-emergent AEs (TEAEs) was reported in 22 (68.8%) of the 31 subjects. Seven TEAEs occurred in 6 subjects (19.4%) in the intervention R1, and 8 TEAEs occurred in 7 subjects (25.5%) in the intervention R2. Ten TEAEs occurred in 7 subjects (23.3%) in the intervention T, and 17 TEAEs occurred in 15 subjects (51.7%) in the intervention V. Two subjects discontinued the study due to AEs (neutropenia for 1 subject in the intervention T, blood creatine phosphokinase increased for 1subject in the intervention R). Among the 42 TEAEs, 3 TEAEs were moderate, and 1 TEAE (blood creatine phosphokinase increased) was severe in intensity while the others were mild. Of the 42 TEAEs, 33 cases were evaluated as possibly related to the intervention.

**FIGURE 4:**
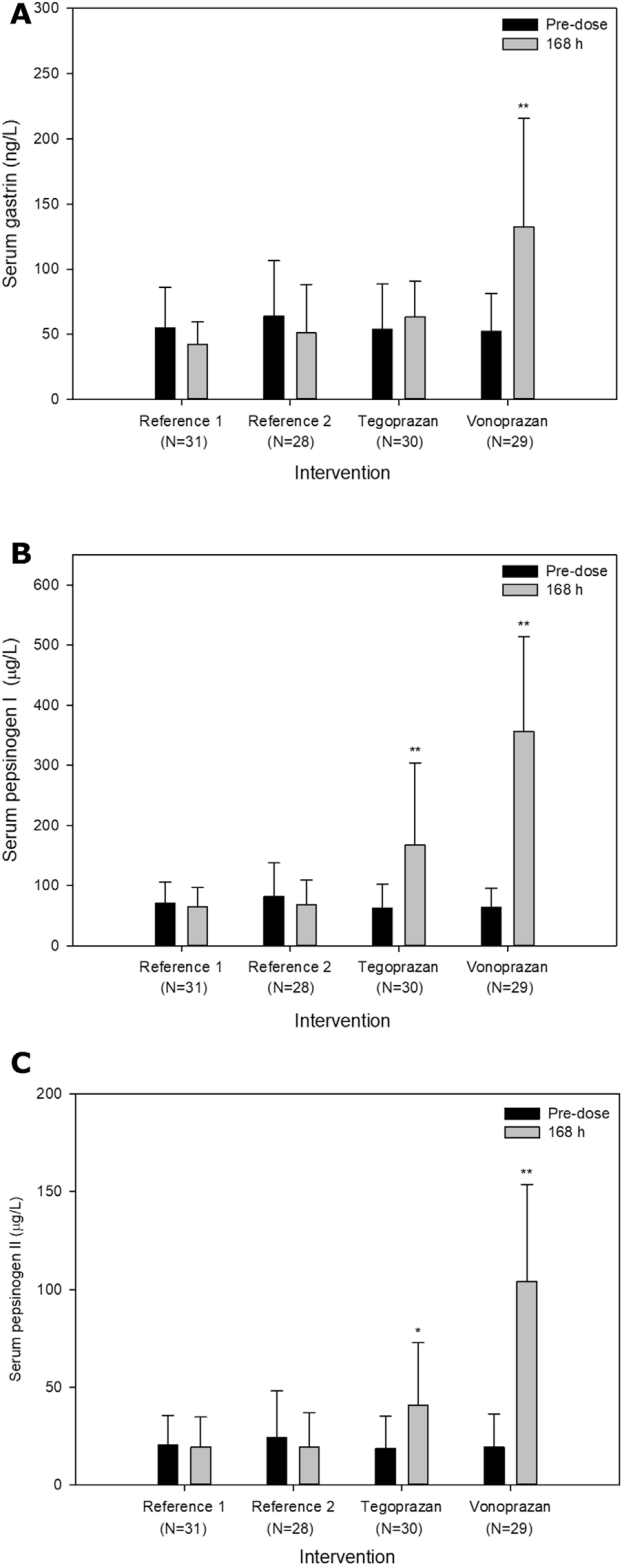
Mean serum **(A)** gastrin levels, **(B)** pepsinogen I and **(C)** II levels pre-dose and 24 h after oral administration of atorvastatin alone for 7 days (Reference) and co-administered with tegoprazan (Tegoprazan) or vonoprazan (Vonoprazan) for 7 days. Error bars represent standard errors. The increase of serum levels in each intervention was compared to the intervention R. P-values were adjusted by Tukey-Kramer method; *p < 0.05; **p < 0.001.

Frequently reported TEAEs were hypergastrinemia (6 cases), blood bilirubin increased (6 cases), and pyuria (5 cases). Hypergastrinemia, defined by a serum gastrin level greater than 200 ng/L, only occurred when atorvastatin was co-administered with vonoprazan. The serum gastrin, pepsinogen I and II levels overall increased after the administration of tegoprazan or vonoprazan compared to the baseline (Figure 4). The increase of mean serum gastrin after the co-administration of vonoprazan was significantly different compared to other interventions (T vs. V, *p* < 0.001; R vs. V, *p* < 0.001). Additionally, there were significant differences in the extent of the increase in the serum pepsinogen I and II between vonoprazan and tegoprazan (*p* < 0.001 for pepsinogen I; *p* < 0.001 for pepsinogen II). Meanwhile, the changes of LDL-C were not significantly different between the interventions (R vs. T, *p* = 0.347; R vs. V, *p* = 0.872; T vs. V, *p* = 0.858).

The number of subjects with TEAEs when atorvastatin was administered alone (6 subjects in Reference 1 and 7 subjects in Reference 2) was similar to when atorvastatin was co-administered with tegoprazan (7 subjects). However, when atorvastatin was co-administered with vonoprazan, more subjects (15 subjects) reported TEAEs than those when atorvastatin was administered alone (Table 3). There were no clinically significant changes in vital signs, physical examinations, and 12-lead ECGs.

**TABLE 3:** Summary of Treatment-emergent adverse events (TEAEs) reported by two or more subjects after oral administration of atorvastatin for 7 days administered alone and co-administered with tegoprazan or vonoprazan.

Preferred term	Reference 1 (N = 31)	Reference 2 (N = 28)	Tegoprazan (N = 30)	Vonoprazan (N = 29)
Blood bilirubin increased	1 (3.2) [1]	2 (7.1) [2]	2 (6.7) [2]	1 (3.4) [1]
Hypergastrinaemia	—	—	—	6 (20.7) [6]
Pyuria	1 (3.2) [1]	1 (3.6) [1]	1 (3.3) [1]	2 (6.9) [2]
Neutrophil count decreased	—	—	1 (3.3) [1]	2 (6.9) [2]
Dyspepsia	—	—	—	2 (6.9) [2]
Headache	—	—	—	2 (6.9) [2]
Anaemia	1 (3.2) [1]	1 (3.6) [1]	—	—
Ocular discomfort	1 (3.2) [1]	—	1 (3.3) [1]	—

Notes: Reference 1, the first intervention of atorvastatin administered alone; Reference 2, the second intervention of atorvastatin administered alone; Tegoprazan, co-administration of atorvastatin and tegoprazan once a day for 7 days; Vonoprazan, co-administration of atorvastatin and tegoprazan once a day for 7 days.

Values are presented as number of subjects (percentage of subjects with AEs) [number of events].

## Discussion

This study investigated whether the concomitant administration of atorvastatin with P-CABs, tegoprazan or vonoprazan, influences the PK of atorvastatin and safety profile. Atorvastatin was known as a highly variable drug with a within-subject CV of more than 30%. Based on the previous replicate crossover studies of atorvastatin, the within-subject CV of C_max_ and AUC were 39–45% and 17–23%, respectively (Hwang et al., 2020; Kim et al., 2020). In the case of highly variable drugs such as atorvastatin, some regulatory agencies recommend a full or partial replicated crossover design (US Food and Drug Administration, 2001). Therefore, this study was designed as a partial replicated crossover study where subjects received intervention R twice and received intervention T and V once. In this study, the within-subject CV of C_ss,max_ and AUC_τ_ of atorvastatin were 33.6 and 11% (data not shown), slightly smaller than those of previous studies (38–44.1% for C_max_ and 16.7–15.5% for AUC) (Hwang et al., 2020; Kim et al., 2020). This difference might be mainly due to multiple doses of atorvastatin for 7 days, reducing the within-subject variability. The high variability in the plasma concentrations of atorvastatin, especially in the absorption phase, was also observed in other studies with similar standard deviations (Hwang et al., 2020; Kim et al., 2020).

Atorvastatin is orally administered in the hydroxy acid ring form and interconverted to the inactive lactone form, and both forms are metabolized to hydroxylated forms by CYP3A4 (Lennernäs, 2003; Hermann et al., 2006). In this study, co-administration of atorvastatin with vonoprazan increased the systemic exposure of atorvastatin and decreased the systemic exposure of 2-hydroxyatorvastatin, resulting in a significant decrease in the metabolic ratio of 2-hydroxyatorvastatin. When atorvastatin was co-administered with vonoprazan, 2-hydroxyatorvastatin was formed relatively slower than when atorvastatin was administered alone. In addition, the slopes of the elimination phase in the log-linear profiles were similar among all the intervention groups. Considering the high permeability of atorvastatin, the changes in the PK of atorvastatin reflected the changes in the activity of CYP3A4 which is independent of the permeability. The IC50 values of CYP3A4 for tegoprazan and vonoprazan were >30 and 29 μM, respectively, and the expected plasma concentrations of tegoprazan and vonoprazan when administered at clinical doses are 25-fold lower than the IC50 (Nishihara et al., 2019; HK inno.N Corp). However, when vonoprazan 20 mg is orally administered, the intestinal luminal concentration of vonoprazan is estimated to be approximately 232 μM that is 8-fold higher than the IC50 for vonoprazan (US Food and Drug Administration, 2020b). Vonoprazan with high luminal concentrations may inhibit the intestinal CYP3A4, thus increasing the systemic exposure of orally administered atorvastatin. Similarly, some clinical studies revealed that concurrent use of statins with CYP3A4 inhibitors, such as protease inhibitors and diltiazem, inhibited the intestinal CYP3A4 increasing systemic exposure of atorvastatin or simvastatin (W Jacobsen, 2000; Fichtenbaum CJ et al., 2002). These results indicated that vonoprazan would inhibit the intestinal CYP3A4, not the hepatic CYP3A4.

The atorvastatin lactone form is inactive in the effect of lowering lipid (Hermann et al., 2006). However, a recent study suggested that atorvastatin lactone might contribute to the occurrence of muscular adverse events such as myopathy (Hermann et al., 2006). Therefore, the effect of co-administration with tegoprazan or vonoprazan on the systemic exposure of atorvastatin lactone should be considered. The atorvastatin lactone is formed by uridine diphosphate–glucuronosyltransferase (UGT) 1A1 and UGT1A3 and sequentially metabolized to 2-, 4-hydroxyatorvastatin lactone by CYP3A4 (Lennernäs, 2003; Hermann et al., 2006). In this study, the systemic exposure of atorvastatin lactone significantly increased when atorvastatin was co-administered with vonoprazan. This increase implies that vonoprazan could inhibit CYP3A4 which involves the formation of 2-, 4-hydroxyatorvastatin lactone (Hermann et al., 2006). In contrast to 2-hydroxyatorvastatin, the metabolic ratio of atorvastatin lactone was similar between all intervention groups. According to this study, it is confirmed that vonoprazan and tegoprazan do not affect UGT1A1 and UGT1A3.

The liver-specific transporter OATP1B1, uptaking endogenous compounds and drugs into hepatocytes, plays an important role in eliminating atorvastatin and its metabolites (Lau et al., 2007). The endogenous bile acid derivative bilirubin is one of the substrates of OATP1B1 like atorvastatin (Lau et al., 2007). Co-administration of atorvastatin with OATP1B1 inhibitors increased the systemic exposure of atorvastatin and induced transient hyperbilirubinemia (Lau et al., 2007). Although tegoprazan showed the inhibitory activity on some OATP1B1 substrates in a non-clinical study, the systemic exposure of the substrates would not significantly increase considering the maximum concentration of tegoprazan at clinical doses (HK inno.N Corp). In this study, when atorvastatin was co-administered with tegoprazan, the C_ss,max_ and AUC_τ_ of atorvastatin increased by 9 and 3%, respectively. These increases were not clinically significant since none of them were statistically significant and the within-subject CVs of the C_ss,max_ and AUC_τ_ of atorvastatin are known to be high (Hwang et al., 2020; Kim et al., 2020). Thus tegoprazan would not have any effect on OATP1B1 at clinical dose. Also, the *in vitro* study showed that vonoprazan did not inhibit OATP1B1 (Pharmaceuticals and Medical Devices Agency, 2014). The increase in the blood bilirubin in this study was similar regardless of whether atorvastatin was administered alone or in combination with P-CABs. Accordingly, the increase of the systemic exposure of atorvastatin co-administered with vonoprazan in this study resulted from inhibiting CYP3A4, not attributed to the inhibition of OATP1B1.

It is important whether the changes of PK by drug-drug interactions affect the efficacy. The dose-response of atorvastatin generally shows a log-linear profile, but the PK is not directly correlated with the LDL-C lowering effect of atorvastatin (Lennernäs, 2003). As expected, the changes of serum LDL-C levels were not statistically different between the interventions in this study. However, further studies are needed to determine the impact of these interactions on LDL-C levels in patients with dyslipidemia since this study was conducted in healthy adults.

Some studies showed that serum gastrin, pepsinogen I and II increased after P-CAB treatment (Inatomi et al., 2016), and hypergastrinemia could be observed frequently when PPIs, including P-CABs, are administered (Mori and Suzuki, 2019; Cho et al., 2020). Given the mechanism of action of the PPIs and subsequent physiological feedback increasing serum gastrin to lower gastric pH, hypergastrinemia can be predictable. Nonetheless, the serum gastrin increased only 1.2 times higher when atorvastatin was co-administered with tegoprazan. Similarly, other studies reported that tegoprazan as well as other gastric acid inhibitors did not compensatively increase the serum gastrin level (Sunwoo et al., 2020; Cho et al., 2020). The mechanism by which tegoprazan does not increase the serum gastrin level has not yet been elucidated.

The gastrin stimulates the pepsinogen release from chief cells and the increase of pepsinogen I and II is predictable (Qian et al., 1993). In this study, the levels of pepsinogen I and II significantly increased when atorvastatin was co-administered with tegoprazan or vonoprazan compared to when atorvastatin was administered alone. However, when atorvastatin was co-administered with vonoprazan, the increases in serum pepsinogen I and II were much greater than those when atorvastatin was co-administered with tegoprazan (5.6 and 5.4 times with vonoprazan, respectively; 2.7 and 2.2 times with tegoprazan). Although the serum gastrin level did not significantly increase when atorvastatin was co-administered with tegoprazan, the serum pepsinogen I and II level could increase since the release of pepsinogen is effectively stimulated by other factors such as cholecystokinin.

The increased blood bilirubin was also frequently observed in this study. Regardless of whether tegoprazan or vonoprazan was co-administered, this might be associated with the administration of atorvastatin. Although the incidence of TEAEs between those of atorvastatin administered alone and co-administered with tegoprazan was similar, more subjects reported TEAEs when atorvastatin was co-administered with vonoprazan. Except for the hypergastrinemia, the safety and tolerability profiles were similar among the interventions. Myopathy, which might progress to life-threatening rhabdomyolysis, is one of the remarkable adverse effects of atorvastatin (Lennernäs, 2003). Although the mechanism of statin-induced muscle adverse events is not fully understood, the risk of the adverse events is thought to be associated with the systemic exposure of statin, especially atorvastatin lactone and 4-hydroxyatorvastatin (Hermann et al., 2006). The co-administration of CYP3A4 inhibitors with atorvastatin showed a higher risk of muscular adverse events such as myopathy and rhabdomyolysis (Hermann et al., 2006). Therefore, the further assessment that the co-administration of atorvastatin with vonoprazan potentially inhibits intestinal CYP3A4 should be conducted in terms of the efficacy and the adverse events.

In conclusion, the systemic exposure of atorvastatin and hypergastrinemia incidence increased after multiple doses of atorvastatin co-administered with vonoprazan in healthy subjects. There was no evidence of significant interactions when atorvastatin was co-administered with tegoprazan.

## Data Availability

The original contributions presented in the study are included in the article/Supplementary Material, further inquiries can be directed to the corresponding author.
