# Supplementary material for: Co-Administration of Vonoprazan, Not Tegoprazan, Affects the Pharmacokinetics of Atorvastatin in Healthy Male Subjects
Source: Front Pharmacol. 2021 Nov 11;12:754849. doi: 10.3389/fphar.2021.754849 (PMC8632694; doi:10.3389/fphar.2021.754849)
Supplement: Supplementary file 1 [file Table1.DOCX]

**Supplementary Table 1. Comparison of pharmacokinetic parameters of metabolites of atorvastatin (2-hydroxy atorvastatin, atorvastatin lactone) after oral doses of atorvastatin administered alone and co-administered with tegoprazan or vonoprazan**

|  | **Parameters** | **Geometric least squares mean** | | **GMR**  **(90% CI)** | **Geometric least squares mean** | | **GMR**  **(90% CI)** |
| --- | --- | --- | --- | --- | --- | --- | --- |
|  |  | **With Tegoprazan**  **(N=28)** | **Atorvastatin alone**  **(N=28 for each intervention)** |  | **With Vonoprazan**  **(N=28)** | **Atorvastatin alone**  **(N=28 for each intervention)** |  |
| 2-hydroxy atorvastatin | C_ss,max_ (μg/L) | 23.8 | 22.5 | 1.06  (0.95-1.17) | 15.91 | 22.7 | 0.7  (0.63-0.78)^*^ |
|  | AUC_τ_ (h·μg/L) | 144.8 | 137.9 | 1.05  (1.00-1.10) | 125.1 | 138.2 | 0.91  (0.87-0.95) |
| Atorvastatin lactone | C_ss,max_ (μg/L) | 7.2 | 6.9 | 1.04  (0.97-1.12) | 9.1 | 6.9 | 1.32  (1.24-1.41)^*^ |
|  | AUC_τ_ (h·μg/L) | 52.1 | 52.1 | 1  (0.96-1.04) | 67.3 | 52.1 | 1.29  (1.23-1.35)^*^ |
| Tegoprazan, co-administration of atorvastatin and tegoprazan once a day for 7 days; Vonoprazan, co-administration of atorvastatin and tegoprazan once a day for 7 days.  GMR is calculated as a ratio of atorvastatin co-administered with tegoprazan or vonoprazan to administered alone.  * According to the equivalence test, if the 90% confidence interval is not included in the predefined equivalence range (0.8 – 1.25), it is not considered equivalent.  Abbreviations: AUC_τ_, area under the curve over a dosing interval at steady state; C_ss,max_, maximum concentration at steady state; CI, confidence interval; GMR, the geometric mean ratio | | | | | | | |
